# Expression of estrogen and progesterone receptor genes in endometrium, myometrium and vagina of postmenopausal women treated with estriol

**DOI:** 10.1590/S1516-31802009000300004

**Published:** 2009-10-06

**Authors:** Magdalena Bryś, Krzysztof Szyłło, Hanna Romanowicz-Makowska, Zbigniew Dobrowolski, Izabela Masłowska, Wanda Krajewska

**Affiliations:** 1 PhD. Associate professor and project leader, Department of Cytobiochemistry, University of Lodz, Lodz, Poland.; 2 MD. Assistant professor, Department of Surgical Gynecology, Research Institute of the Polish Mothers’ Memorial Hospital, Lodz, Poland.; 3 MD. Pathologist, Department of Pathology, Research Institute of the Polish Mothers’ Memorial Hospital, Lodz, Poland.; 4 MD. Gynecologist, Department of Surgical Gynecology, Research Institute of the Polish Mothers’ Memorial Hospital, Lodz, Poland.; 5 PhD. Researcher, Department of Cytobiochemistry, University of Lodz, Lodz, Poland.; 6 PhD. Professor and supervisor, Department of Cytobiochemistry, University of Lodz, Lodz, Poland.

**Keywords:** Receptors, estrogen, Receptors, progesterone, Estriol, Hormone replacement therapy, Reverse transcriptase polymerase chain reaction, Receptores estrogénicos, Receptores de progesterona, Estriol, Terapia de reemplazo de hormonas, Reacción en cadena de la polimerasa de transcriptasa inversa

## Abstract

**CONTEXT AND OBJECTIVE:**

Estriol is an estrogen with considerably weaker stimulatory effects on endometrial proliferation than estradiol. A study was conducted to determine the level of estrogen receptors (ERs) and progesterone receptors (PRs) in women who received 14-day vaginal estriol therapy, compared with those who did not receive this therapy. ER and PR gene expression was analyzed in the endometrium, myometrium and vagina of postmenopausal women treated with estriol.

**DESIGN AND SETTING:**

Analytical cross-sectional study, at the Research Institute of the Polish Mothers’ Memorial Hospital, Lodz, Poland.

**METHODS:**

Twenty-seven postmenopausal women (57-74 years of age) were included in the study. All of them were waiting for *per vaginam* hysterectomy or plastic surgery on the vagina and perineum because of uterine prolapse. ER and PR gene expression was determined by means of the technique of reverse transcription polymerase chain reaction (RT-PCR).

**RESULTS:**

In the estriol-treated patients, in comparison with the control group, a significant increase in ER gene expression was observed in the endometrium and vagina, while enhanced PR gene expression was found in the endometrium. However, under histological examination of the endometrium, estrogen stimulation of low and medium degree was diagnosed for 21.4% and 14.3% of the estriol-treated women, respectively.

**CONCLUSION:**

The results obtained suggest that the women who received 14 days of treatment with vaginal estriol had higher ER and PR mRNA levels. No difference between these groups regarding endometrial proliferation was observed.

## RESUMÉN

**CONTEXTO Y OBJETIVO:**

El estriol es un estrógeno con un efecto estimulatorio bastante más débil sobre la proliferación endometrial que el estradiol. Se realizó un estudio para determinar los efectos de una terapia vaginal de 14 días con estriol, sobre el nivel de receptores de estrógeno (ER) y receptores de progesterona (PR), comparado con mujeres sin esa terapia. La expresión de los genes de ER y PR se analizó en el endometrio, miometrio y vagina de mujeres posmenopáusicas tratadas con estriol.

**DISEÑO Y UBICACIÓN:**

Estudio Transversal analítico, en el Instituto de Investigación del Hospital de la Madre Polaca en Lodz, Polonia.

**MÉTODOS:**

Se incluyeron veintisiete mujeres posmenopáusicas (de 57 a 74 años) en el estudio. Todas ellas estaban en espera de una histerectomía *per vaginam* o de cirugía plástica de la vagina y del perineo debido a un prolapso del útero. La expresión de genes de los receptores ER y PR se estableció por la técnica de RT-PCR.

**RESULTS:**

En las pacientes tratadas con estriol en comparación con el grupo de control, se observó un aumento significativo de la expresión del gen de ER en el endometrio y la vagina, mientras que un aumento de la expresión del gen de PR se encontró en el endometrio. De todas formas, en el examen histológico del endometrio, se diagnosticó estimulación estrogénica de bajo y medio grado en el 21.4% y en el 14.3% de las mujeres tratadas con estriol, respectivamente.

**CONCLUSIONES:**

Los resultados obtenidos sugieren que un tratamiento de 14 días con estriol en pacientes, aunque aumentan el nivel de ER y de PR mRNA, tiene muy poco o ningún efecto sobre la proliferación endometrial.

## INTRODUCTION

Progress in medicine and general improvement in socioeconomic conditions have resulted in longer lifetimes and an increased population of postmenopausal women. The postmenopausal period is a natural period in women’s lives during which the processes of ageing take place. Hormonal and psychosomatic disturbances intensify. With regard to hormones, hypoestrogenism dominates, together with increased levels of gonadotropins and a drop in the level of androgens.^1^

Estrogens play a crucial role in regulating the physiology of breast tissue and the endometrium.^2^^,^^3^^,^^4^ Furthermore, estrogen has been implicated in the initiation and progression of neoplasms of these tissues. Estrogens mediate their effects through estrogen receptor isoforms and isotypes.^5^ Both estrogen receptors (ERs) and progesterone receptors (PRs) have two subtypes, i.e. ER-alpha and beta, and PR-A and B, respectively.^6^ These subtypes differ in function and expression, and recent reports have correlated changes in the normal proportions of these isoforms with neoplastic states. The potential clinical relevance of differential ER-isotype expression has also been discussed with regard to antihormonal therapy.^7^

Estrogens have neuroprotective and antiapoptotic properties. However, the issue of what involvement the ER-dependent genomic pathway has in these effects still remains controversial.^8^^,^^9^

ERs and PRs are members of the nuclear receptor superfamily of ligand-dependent transcription factors. It has been found that, in the absence of ligands, ERs and PRs located in target cells are associated with the complex of heat shock proteins and chaperones. Upon ligand binding, the receptors undergo conformational changes that enable spontaneous dimerization. This event facilitates the interaction of the receptors with specific DNA enhancer sequences located within the regulatory regions of target genes that regulate estrogen or progesterone-responsive genes.^10^^,^^11^^,^^12^^,^^13^

ER and PR gene expression may be regulated by estriol (E3) in the uterus and other reproductive tissues such as the vagina. Estriol derivatives are commonly used in therapy. Estriol has been considered to be an antagonist of estradiol.^14^^,^^15^^,^^16^^,^^17^

The data so far available concerning estriol action on the endometrium, myometrium, vagina and breast are ambiguous, and they provide contradictory information.^18^^,^^19^^,^^20^^,^^21^ It seems that differences in the affinity, specificity and concentrations of ERs in individual tissues are of much greater importance, together with changes in effector tissues that lead to different responses.

## OBJECTIVE

In the present study, the influence of 14-day vaginal estriol administration on ER and PR messenger ribonucleic acid (mRNA) expression in the endometrium, myometrium and vagina was investigated within the context of hormonal endometrial stimulation.

## METHODS

### Patients

Twenty-seven postmenopausal patients were included in the study. All of them were waiting for *per vaginam* hysterectomy or plastic surgery on the vagina and perineum because of uterine prolapse. All the patients were treated and operated in the Department of Surgical Gynecology, Research Institute of the Polish Mothers’ Memorial Hospital between 2000 and 2002. Thirteen patients were singled out as the control group. The others were treated daily with 0.5 mg of estriol (Ovestin® cream, made by Organon, Poland, applied vaginally with a calibrated applicator) for two weeks before the operation. The last dose of the drug was administered around 48 hours before the operation. The presence of oncological diseases was ruled out by means of anamnesis, physical examination and laboratory investigation. Before the operation, all the patients underwent fractionated uterine curettage prior to hormonal treatment. They also underwent endometrial ultrasonography before and after the treatment. Postmenopausal status was determined on the basis of serum follicle stimulating hormone (FSH) levels. Moreover, for each patient, a cytohormonal examination was conducted, and the maturation index was determined before and after estriol treatment.

The operations were performed in accordance with standard procedures. During the operation, immediately after the uterus and a small sleeve of vaginal tissue were removed, these tissues were placed in ice and transferred to the Department of Pathology. The body of the uterus was dissected and samples of the endometrium and myometrium were taken. These, together with samples of vaginal tissues, were stored in three separate containers at - 70 °C until the biochemical investigation had been completed. Other tissue fragments were placed in a solution of buffered formalin and then subjected to pathological examination, in accordance with standard procedures. The samples taken from the uterus and the vagina were examined by an experienced pathologist under an optical microscope.

### RNA extraction and reverse transcription

Total RNA was extracted from the endometrial, myometrial and vaginal samples of the estriol-treated patients and control group by means of the acid guanidinium thiocyanate-phenol-chloroform method (all chemicals supplied by Sigma-Aldrich, United States). It was quantified by means of spectrophotometry at 260 nm. RNA with a 260/280 nm ratio within the range 1.8-2.0 was considered high quality.^22^ First-strand complementary deoxyribonucleic acid (cDNA) was synthesized from each RNA pool using the RNA polymerase chain reaction (PCR) kit version 2.1 (Takara Shuzo Co. Ltd, Japan), in accordance with the manufacturer’s directions. Briefly, 1 µg of RNA was combined with 2.5 pmol of oligo dT-adaptor primer, 4 µl of 25 mM MgCl_2_, 10 x RNA PCR buffer, 2 µl of 10 mM dNTP mixture, 20 units of RNase inhibitor, five units of AMV Reverse Transcriptase XL and RNase-free water to a total volume of 20 µl. The mixture was placed in a thermal cycler and incubated using the following condition: 52 °C for 20 minutes, 99 °C for five minutes, and 5 °C for five minutes.

### PCR amplification

To perform the reverse transcription polymerase chain reaction (RT-PCR), we followed the protocol of Chevillard et al.^23^ For each cDNA species of interest and for that of a “housekeeping” gene, we first determined the range of cycles over which the species should be examined. The linear amplification range for each gene was tested on the adjusted cDNA. The ER and PR gene expressions were determined following co-amplification and normalization by means of an internal control sequence, i.e. using b2-microglobulin (b2m). The specific primers for RT-PCR are shown in Table 1.

The thermal cycling parameters consisted of 35 rounds of 30 seconds at 94 °C, 50 seconds at 58 °C and 20 seconds at 72 °C. The sizes of the amplified fragments were 239, 205 and 165 bp for ERs, PRs and b2m, respectively. After amplification, 10 µl of the PCR products were combined with 1 µl of gel loading buffer. This mixture was electrophoresed on 5% (ER) and 7% (PR) polyacrylamide gel (acrylamide/bisacrylamide (30/2), 10% ammonium peroxydisulfate and 0.075% tetramethyl ethylenediamine), at 80 V for three hours in Tris-borate, EDTA, pH 8.0 (TBE) buffer (all chemicals supplied by Sigma-Aldrich, United States). The gel was silver stained.^24^

To rule out contamination of genomic DNA as a source for amplified products, each reaction was also carried out without reverse transcriptase. In addition, a control without the template was run for both the RT and PCR stages, for each of the primer sets, and none of these showed any visible PCR products.

**Table 1.** Specific primers for quantitative reverse transcription polymerase chain reaction (RT-PCR)

*AR* ^*u*^	5’-AGCTACTCCGGACCTTACG-3’
*AR* ^*d*^	5’-AGGTGCCATGGGAGGGTTAG-3’
*GAPDH* ^*u*^	5’-GCCACATCGCTCAGACACCA-3’
*GAPDH* ^*d*^	5’-GATGACCCTTTTGGCTCCCC-3’
*ER-*a^*u*^	5’-ACTCGCTACTGTGCAGTGTGCAATG-3’
*ER-*a^*d*^	5’-CCTCTTCGGTCTTTTCGTATCCCAC-3’
*PR-A* ^*u*^	5’-GGCAGCACAACTACTTATGTGC-3’
*PR-A* ^*d*^	5’-TCATTTGGAACGCCCACT-3’
b*2m* ^*u*^	5’-CATCCAGCGTACTCCAAAGA-3’
b*2m* ^*d*^	5’-GACAAGTCTGAATGCTCCAC-3’

superscript u = upstream primer; superscript d = downstream primer. AR = androgen receptor; GAPDH = glyceraldehyde 3-phosphate dehydrogenase; ER = estrogen; PR-A = progesterone receptor-A; ß2m = beta-2 microglobulin.

### Qualitative and quantitative estimations of electropherograms

For qualitative and quantitative analysis of the silver nitrate stained gels, a video densitometer (Biotec-Fischer, Reiskirchen, Germany) with the software program Gel-Pro® Analyzer 3.0 (Media Cybernetics, United States) was used. All RT-PCR reactions were done three times for each sample. The integrated optical density (IOD) of the bands on digitized images was measured. ER or PR gene expression was expressed as the ratio of ER or PR over b2m. The ratio between the PCR amplified gene and the amplified standard was obtained for each sample. A conventional cutoff of 15 fmol/mg protein for ER status corresponded to an ER/b2m ratio of 0.5 and, according to the regression equation, the conventional cutoff of 15 fmol/mg protein for PR status corresponded to a PR/b2m ratio of 0.03.^23^

### Histological examination of the endometrium

Each patient’s endometrium was examined under an optical microscope for histological evidence of estrogen stimulation. When exposed to excessive long-term estrogen action, the endometrium normally reacts by stimulating proliferation processes, and thus the following criteria were taken into consideration: 1) glandular surface/stroma ratio; 2) distribution, size and shape of glandular tubes; 3) cell distribution in tubes; 4) cell stratification in tubes; 5) mitotic activity; and 6) cellular atypia.

The endometrial samples evaluated were scored within a possible range of 0-6 points; 1-2 points signified weak estrogen action; 3-4 points, medium-strength action; and 5-6 points, strong action.

### Statistical analysis

None of the parameters recorded in the tumor material passed the test for normal distribution (Kolmogorov-Smirnov test). Hence, nonparametric statistical tests were used throughout, for analyzing the results. P-values < 0.05 were considered to be significant.

## RESULTS

### ER and PR mRNA expression

**Figure 1.**
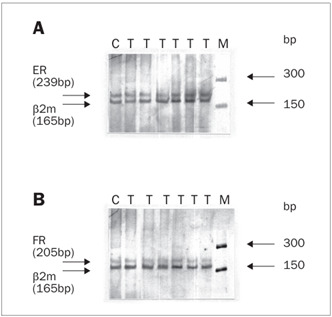
Analysis of the estrogen receptor (ER) (A) and progesterone receptor (PR) (B) messenger ribonucleic acid (mRNA) expression in the endometrium, myometrium and vagina of women treated with estriol, taking the ß2-microglobulin (ß2-m) levels as the internal standard. C = tissue from control (untreated) patient; T = tissue from estriol-treated patient; M = molecular weight ladder marker for 50-2000 base pairs (bp) (Sigma, St. Louis, United States).

ER and PR gene expression in the endometrium, myometrium and vagina of women treated with estriol, in comparison with the control group, was investigated by means of RT-PCR. A representative electropherogram from the RT-PCR analysis of ER and PR gene expression is shown in Figure 1.

In the group of estriol-treated women, ER mRNA was detected in 85.71% (12/14), 78.57% (11/14) and 100% (14/14) of the endometrial, myometrial and vaginal specimens, respectively. In the control group, ER mRNA expression was observed in 61.54 % (8/13) of the endometrial, 61.54% (8/13) of the myometrial and 69.23% (9/13) of the vaginal specimens.

ER mRNA was found to be overexpressed in 78.57% (11/14), 42.86% (6/14) and 92.86% (13/14) of the endometrial, myometrial and vaginal samples obtained from the estriol-treated women, respectively. Statistically significant differences (Mann-Whitney U test, P < 0.001) were recorded between ER gene expression in the endometrium and vagina of the estriol-treated women and control group (Table 2).

In the tissues obtained from estriol-treated women, PR mRNA was detected in 85.71% (12/14) of the endometrial, 85.71% (12/14) of the myometrial and 50% (7/14) of the vaginal samples. In the group of control tissues, PR mRNA expression was observed in 46.15% (6/13), 53.85% (7/13) and 46.15% (6/13) of the endometrial, myometrial and vaginal samples, respectively.

PR mRNA was overexpressed in 50% (7/14) of the endometrial, 42.86% (6/14) of the myometrial and 21.43% (3/14) of the vaginal samples obtained from the estriol-treated women. Statistically significant differences (Mann-Whitney U test, P < 0.001) were noted between PR gene expression levels in the endometrium of the estriol-treated women and control group (Table 2).

**Table 2.** Relative estrogen and progesterone receptor messenger ribonucleic acid (mRNA) levels

Tissues	Patients	
	Estriol-treated group n = 14	Control group n = 13
	ER (IOD _ER_/IOD_b2m_)	
Endometrium*	1.24 ± 0.04	0.57 ± 0.06
Myometrium	0.64 ± 0.06	0.58 ± 0.07
Vagina*	0.97 ± 0.04	0.48 ± 0.04
	PR (IOD _PR_/IOD_b2m_)	
Endometrium*	0.079 ± 0.008	0.032 ± 0.003
Myometrium	0.057 ± 0.004	0.036 ± 0.004
Vagina	0.050 ± 0.011	0.061 ± 0.006

*statistically significant differences. ER = estrogen receptor; PR = progesterone receptor; IOD = integrated optical density; β2m = beta-2 microglobulin

### Cytohormonal examination

All of the estriol-treated patients showed a higher maturation index, with a shift towards superficial cells. The smears from the control group were atrophic. Histological evaluation showed atrophic features for almost all the patients included in the study. In only one case, ultrasonography (USG) showed an expanded endometrium (12.5 mm). However, histopathological examination did not provide any evidence of endometrial proliferation. After the estriol treatment, a small degree of proliferation was detected in 5/14 cases (36%) at the postoperative assessment. The endometrium of these five patients showed symptoms of hormonal stimulation, with three low-degree cases (21.4%) and two medium-degree cases (14.3%). Out of the 14 estriol-treated patients, nine did not show estrogen stimulation of the endometrium: seven cases were defined as atrophic and two as typical. The endometrium from the control group showed no evidence of estrogen stimulation. It was defined as atrophic in 12 cases, and typical in one case (Table 3).

**Table 3.** Histological analysis on endometrium

Patients	Endometrial evaluation			
	No stimulation	Low degree of stimulation	Medium degree of stimulation	High degree of stimulation
Estriol-treated group	9/14 (64.3%)	3/14 (21.4%)	2/14 (14.3%)	0
Control group	13/13 (100%)	-	-	-

## DISCUSSION

Regulation of gene transcription is central both to tissue specific-gene expression and to regulation of gene activity in response to specific hormonal stimuli. In order to produce effects, transcription factors will require the ability to bind to DNA and then to influence transcription either positively or negatively.^25^^,^^26^

ERs and PRs as transcription factors can be regulated at two levels, i.e. the levels of transcription factor synthesis and transcription factor activity. Furthermore, they are regulated through synthesis in one particular tissue or cell type and not in other tissues.^27^^,^^28^^,^^29^

Although the regulation of transcription factor synthesis is an important control point, it cannot be the only regulatory mechanism controlling transcription factor activity. If this were the case, enhanced synthesis of a transcription factor in response to a particular stimulus would be controlled by enhanced transcription of its corresponding gene, which in turn would require *de novo* synthesis of further transcription factors, thus resulting in the need for new transcription of these genes, and so on.^25^

In a series of experiments, we demonstrated the expression levels of ER and PR mRNA in the endometrium, myometrium and vagina of postmenopausal women treated with estriol. Our data showed differences between the three tissues studied. Significantly higher ER and PR mRNA levels were found in the endometrium of estriol-treated women, compared with the control group. In the case of vaginal tissue, only ER gene expression was enhanced after estriol therapy. There were no statistically significant differences in ER and PR mRNA levels between the myometrial tissues obtained from the estriol-treated women and control patients. It was observed that the endometrium and vagina were sensitive to estriol stimuli, but the precise mechanism of this phenomenon is unknown. Although the general mechanism through which estrogens and progestins increase their activity in the female reproductive system is well established, the ways in which transcriptional regulation takes place and steroid receptors act remain elusive.

ERs have at least two regions that are required for maximum transcriptional activity: activating function-1 and 2 (AF-1 and AF-2). These AFs have been shown to function in a cell-specific manner and it has been found that the requirement for these activation functions varies depending on the cell and promoter context.^30^ The fact that either AF-1 or AF-2 activity is sufficient for ER action in some contexts, whereas both are required in others, suggests that the role of ERs in different cells and on different promoters is not the same. Thus, it has been proposed that ERs are components of two distinct signaling pathways within the cell: the classical ER/ERE (estrogen receptor/estrogen response element) - mediated signal transduction pathway and/or the non-classical pathway of interaction with AP1 complexes.^31^

It is generally accepted that PR induction is a specific response by an estrogen target tissue to an estrogenic stimulation. PR concentration is hormonally regulated. PRs induced by estrogens and downregulated by progestins.^32^^,^^33^ Analysis of the 5’ flanking region, 5’ untranslated region and exon 1 of rat PR genes has identified four estrogen-responsive enhancers. When linked together as an artificial cassette, these enhancers form a strong estrogen-responsive transcription regulator.^34^

Our results are concordant with the data published by Haaften et al.^21^ These authors measured ERs and PRs through protein levels in patients treated for three weeks with 0.5 mg of estriol daily. They postulated that in human postmenopausal endometrium, ER and PR protein levels increase significantly following vaginal application of estriol. The increases of ER and PR mRNA levels observed in our study may reflect transcription *per se* or posttranscriptional modification, which may alter the stability of the mRNA and/or receptor protein.

The action effected by estriol on the endometrium is unclear. Any stimulation of the endometrium that may occur depends directly on the pharmacokinetic and pharmacodynamic properties of estriol, i.e. the speed of absorption, conjugation and secretion, and on rapid dissociation of nuclear receptors.^35^^,^^36^

Endometrial atrophy prior to treatment had not been assessed by other authors and, in the case of one study, only the results from the progesterone test were taken into consideration.^37^ In a long-term study, Boseli et al. evaluated the endometrium 3, 6, 12, 18 and 24 months after a vaginal estriol preparation in 0.5 mg doses had been applied, and they observed no proliferative changes.^38^ Furthermore, Weiderpass et al. found that vaginal treatment with low-potency estrogen formulations had no impact on the relative risk of endometrial neoplasia.^39^

No proliferative changes were observed in our study after 14 days of estriol application. Only a low degree of endometrial stimulation was noted. The answer to the question of whether 14-day estriol therapy before the operation is safe and effective was affirmative. The therapy was effective, considering that the atrophic condition of the vaginal tissue improved in all of the patients. This was confirmed by the increased maturation index and enhanced ER expression. The healing of the wound was regular for all the patients, and the postoperative period did not exceed nine days. The therapy was safe, considering that none of the patients showed any symptoms of atypical endometrium proliferation, even though their ER and PR mRNA levels were overexpressed. These results are concordant with those of other authors, who demonstrated that noticeable proliferative changes to the endometrium appeared after at least three weeks of administering doses of 0.5 mg of estriol.^21^

## CONCLUSION

The results obtained suggest that although 14 days of estriol treatment enhanced the patients’ ER and PR mRNA levels, it had very little or no effect on the proliferation status of the endometrium.
